# Selection index for beef cattle that maximizes overall growth yet constraining birth weight and other traits

**DOI:** 10.5713/ab.24.0912

**Published:** 2025-08-12

**Authors:** Kenji Togashi, Toshio Watanabe, Atsushi Ogino, Masakazu Shinomiya, Kazuhito Kurogi, Masanobu Nurimoto

**Affiliations:** 1 Livestock Improvement Association of Japan, Maebashi, Japan; 2 Livestock Improvement Association of Japan, Tokyo, Japan

**Keywords:** Birth Weight, Growth Curve, Growth Maximization, Index, Random Regression, Selection Intensity

## Abstract

**Objective:**

Maximizing growth throughout the growth period is a practical goal in the beef industry. We developed a novel selection index, called the maximum growth index, that maximizes the growth throughout the growth process and simultaneously achieves the desired weight gain at designated time points by assigning arbitrary values to selection intensity.

**Methods:**

We used a technique called Lagrange multipliers to maximize overall growth throughout the entire growth process and to restrict weight gains to desired amounts at specific times. We developed a selection index by applying random regression (RR) to the growth curve and using the genomically enhanced breeding values of the RR coefficients as selection index traits.

**Results:**

Examples of the developed index was applied based on assumed data in Japanese Black steers. Selection to maximize growth during the growth period and to moderate birth weight yielded an upwardly convex curve for weight gain during the growth process, and the peak daily gain was greater and earlier than achieved without this selection. Under a selection intensity of 0.5, the index that constrained birth weight to decrease by 2.5 kg achieved the pre-selection final weight 8 weeks earlier than occurred without this selection.

**Conclusion:**

We developed a beef cattle selection index that maximized total weight gain during growth yet constrained birth weight and other traits. The maximum growth index that we developed enables reductions in birth weight concurrent with increases in final weight, thus benefiting the beef industry by increasing final weight and preventing dystocia.

## INTRODUCTION

Previously developed index achieves the desired genetic weight gain at a given time point and simultaneously minimizes increases in inbreeding by minimizing selection intensity; this index is anticipated to support sustainable genetic improvement and yet preserve genetic diversity [1,2]. However, minimized selection intensity is the resulting value required to achieve the desired amount of weight gain at the designated time with the least selection intensity and selection intensity cannot be artificially assigned an arbitrary value in advance. Moreover, the indices developed [1,2] can minimize the intensity of selection but not maximize growth during growth process. Maximizing growth is a primary goal in beef industry. Therefore, a new selection method is needed to maximize growth throughout the growth process while achieving the desired weight gain at the target time point. This new selection index was designated as the maximum growth index.

In beef cattle, birth weight is correlated with dystocia and mortality during the first 24 h of life [3,4], and one strategy to avoid these complications has been to mate heifers with bulls that have low estimated breeding values (EBVs) for birth weight [5]. However, given the positive genetic correlation between birth weight and body weight during growth [6], the offspring of these low-EBV bulls tend to weigh less than their counterparts at the end of fattening. The above biological problem between birth weight and final weight may be solved by the mathematical method of Lagrange multipliers. Development of the selection index utilizing Lagrange multipliers for moderate birth weight, maximizing growth throughout the growth process, and increasing final weight will resolve the above issues and benefit the beef industry.

Random regression (RR) models have been applied for the genetic evaluation of longitudinal data, including growth, lactation, and egg-production curves in poultry [7–11].

In particular, RR models have been applied to analyze the entire fattening process of beef cattle [12–15], and a stage-gain index based on RR coefficients, i.e., Legendre polynomials, that minimized selection intensity was developed for the lactation curves of dairy cattle [10]. Because they include all weights and appropriate covariances, predictions based on RR models are highly accurate [16]. Moreover, the genomic information from RR growth curves to select young animals would concurrently improve genetic growth throughout the growth trajectory and reduce the generation interval. The genomically enhanced breeding values (GEBVs) of the RR coefficients can be treated as selection index traits of the maximum growth index. The maximum growth index could apply to other breeds or longitudinal traits beyond Japanese Black cattle by applying RR models and using the RR coefficients of the RR models based on Legendre polynomials as selection index traits. Although this study uses GEBVs of the RR coefficients, EBV or phenotype itself [2] of the RR coefficients can be treated as selection index traits of the maximum growth index. The objective of this study was to develop an index for maximizing weight gain throughout the growth process while achieving desired weight gains at designated time points by assigning arbitrary values to selection intensity. In particular, we sought to develop an index to maximize overall growth but moderate birth weight in beef cattle.

## MATERIALS AND METHODS

We used a RR model based on Legendre polynomials to develop a genomic index for achieving desired weight gains at specific time points during growth while maximizing the total weight gain during growth (excluding the constrained gains at designated time points). Desired weight gains are achieved by the genetic responses to weight gains at designated time points during growth process after one cycle of selection. For example, the genetic response to body weight at birth is restricted to −2.5 kg in beef cattle. In the following sections, we explain and discuss the mathematical bases of our index and then provide numerical examples to demonstrate the overall approach.

### Maximum growth index

We named our index for achieving desired weight gains at specific time points during growth while maximizing the total weight gain during growth (but excluding constrained gains at designated time points) the “maximum growth index.”

The maximum growth index (*I_GEBV_*) was defined as $I_{GEBV}=\sum_{j=0}^{k-1}b_{j}GEBV_{j}=GEBV_{\overset{\prime }{a}L}^{\prime }b$, where *b_j_* is the index weight for the *j^th^* order of the GEBV Legendre RR coefficient; *GEBV_j_* is the GEBV for the *j^th^* order of the Legendre polynomial RR coefficient (α*_Lj_*) including a constant; *GEBV*_α_*_L_* is a (k×1) column vector containing *GEBV_j_* (*j* = 0,1,..,*k*−1) for Legendre polynomial RR coefficients (α*_L_*); k is the number of Legendre coefficients, *α_L_* = (*α_L_*__0__, *α_L_*__1__, …, *α_L_*_*_k_*_−1__); and *b* is a (k×1) column vector of index weights derived to maximize correlation with total genetic value while satisfying restrictions. Legendre RR was fitted as a growth curve in this study. A previously reported index [1,2] for achieving a desired weight gain while minimizing selection intensity, rather than maximizing total weight gain throughout the growth process, is referred to as the “point gain-index.”

Desired genetic gains (Δ*G_s_*) at s specific times during the fattening process can be described according to BLUP properties [17] as:


$$\Delta G_{s}=cov\left(S\alpha_{L},I_{GEBV}\right)\frac{\bar{i}}{\sigma_{I_{GEBV}}}=cov\left(S\alpha_{L}\;GEBV_{\alpha L}^{\prime }b\right)\frac{\bar{i}}{\sigma_{I_{GEBV}}}=SV_{GEBV_{\alpha L}}b\frac{\bar{i}}{\sigma_{I_{GEBV}}},$$

where *S* is an (s×k) matrix;


$$S=\left[\begin{array}{ccccc} \varphi_{0}\left(t_{1}\right) & \varphi_{1}\left(t_{1}\right) & \varphi_{2}\left(t_{1}\right) & .. & \varphi_{k-1}\left(t_{1}\right)\\ \varphi_{0}\left(t_{2}\right) & \varphi_{1}\left(t_{2}\right) & \varphi_{2}\left(t_{2}\right) & .. & \varphi_{k-1}\left(t_{2}\right)\\ .. & .. & .. & .. & ..\\ \varphi_{0}\left(t_{s}\right) & \varphi_{1}\left(t_{s}\right) & \varphi_{2}\left(t_{s}\right) & .. & \varphi_{k-1}\left(t_{s}\right) \end{array}\right];$$

s is the total number of restrictions in the fattening process; *t_i_* is the standardized time value to the interval between −1 to +1 for the *i^th^* specific time in fattening process for the desired gains (*i* = *1*,..,*s*); *ϕ_j_*(*t_i_*) is the *j^th^* order of Legendre polynomial (*j* = *0*,..,*k*−*1*) evaluated at *t_i_*; Δ*G_S_* is an (s×1) column vector;

Δ*G_s_* = (Δ*G_t_*_1_ Δ*G_t_*_2_ . . Δ*G_ts_*)'; Δ*G_ti_* is the desired genetic gain for the i^th^ specific time during growth; *ī* is the intensity of selection; σ*_IGEBV_* is the standard deviation of the maximum growth index (*I_GEVB_)* and *V_GEBVαL_* is a (k×k) (co)variance matrix of *GEBV*_α_*_L_*.

The vector of the difference in Legendre polynomial coefficients between after and before selection (Δα*_L_* is a [k×1] column vector, i. e., α*_L_* after selection – α*_L_* before selection) can be described according to BLUP properties [17] as


(1) $$\Delta \alpha_{L}=V_{GEBV_{\alpha L}}b\frac{\bar{i}}{\sigma_{I_{GEBV}}}.$$

Furthermore, desired genetic gains (Δ*G_s_*) at s specific times during the fattening process are described as Δ*G_s_* = *S*Δα_L_.

Total genetic value from the beginning to the end of the fattening process (i.e., from the 1^st^ through m^th^ specific times during the growth process) is described as *G_L_*, $G_{L}=\sum_{i=1}^{m}F_{i}\alpha_{L}=F\alpha_{L}$; i = 1, 2, …, *m* time point,


$$\left[\begin{array}{c} F_{1}\\ F_{2}\\ ..\\ F_{m} \end{array}\right]=\left[\begin{array}{ccc} F_{1,0} & \cdots & F_{1,k-1}\\ \vdots & \ddots & \vdots \\ F_{m,0} & \cdots & F_{m,k-1} \end{array}\right]\;\;\;;\;F=\sum_{i=1}^{m}F_{i},$$

where *F* is a (1×k) row vector; and *F_i,j_* = the *j^th^* Legendre polynomial of covariate in the i^th^ specific time.

Let the breeding goal be to maximize the growth throughout the fattening process but excluding specific time points constrained to predetermined weight gains, subject to a restriction of $SV_{GEBV_{\alpha L}}b\frac{\bar{i}}{\sigma_{I_{GEBV}}}=\Delta G_{s}$ associated with a prespecified value of *ī*. This is equivalent to maximizing the correlation (*r*) between *I_GEBV_* and *G_L_* subject to the constraint $SV_{GEBV_{\alpha L}}b\frac{\bar{i}}{\sigma_{I_{GEBV}}}=\Delta G_{s}$.

The method of Lagrange multipliers is widely used to find the maximum or minimum of a function when restrictions are imposed on the variables of the function. The method of Lagrange multipliers gives the following function (*f*):


$$f=r_{I_{GEBV},G_{L}}+\eta^{\prime }\left(SV_{GEBV_{\alpha L}}b\frac{\bar{i}}{\sigma_{I_{GEBV}}}-\Delta G_{s}\right),$$

where *η* is an (s×1) column vector of Lagrange multipliers with elements *η_i_* (i = 1, 2, ..., s).

Because $\text{cov}(G_{L},\;I_{\text{GEBV}}^{\prime })=\text{cov}(F\alpha_{L},\;I_{GEBV}^{\prime })=\text{cov}(F\alpha_{L},GEBV_{\alpha L}^{\prime }b)=FV_{GEBV_{\alpha L}}b,\;\sigma_{I_{GEBV}}^{2}=b^{\prime }V_{GEBV_{\alpha L}}b,\;\sigma_{G_{L}}^{2}=FKF^{\prime }$ and var(*α_L_*) = *K*, the correlation (*r_IGEVB_*_, _*_GL_*) between *I_GEBV_* and *G_L_* can be shown as:


$$r_{I_{GEBV},G_{L}}=\frac{b^{\prime }V_{GEBV_{\alpha L}}F^{\prime }}{\sqrt{b^{\prime }V_{GEBV_{\alpha L}}b}\sqrt{FKF^{\prime }}}.$$

Differentiating the function *f* with respect to *b* and equating the resulting partial derivatives to zeros results in the following equations:


(2) $$I_{k}b+[i\text{S}\frac{1}{\sqrt{b^{\prime }V_{GEBV_{\alpha L}}b}}-\frac{1}{\sqrt{b^{\prime }V_{GEBV_{\alpha L}}b}}b\Delta G_{s}^{\prime }]\eta =F^{\prime }$$

, where *I_k_* is a (k×k) identity matrix.

Differentiating the function *f* with respect to *η* and equating the resulting partial derivatives to zeros results in the following equations:


(3) $$\frac{\sigma f}{\sigma \eta }=\frac{\bar{i}}{\sigma_{I_{GEBV}}}SV_{GEBV_{\alpha L}}b-\Delta G_{s}=0,$$

where, $\sigma_{I_{GEBV}}=\sqrt{b^{\prime }V_{GEBV_{\alpha L}}b}$.

Eqs. 2, 3 can be jointly expressed as the following system of equations:


(4) $$\left[\begin{array}{cc} I_{k} & \frac{\bar{i}}{\sigma_{I_{GEBV}}}S^{\prime }-\frac{1}{\sigma_{I_{GEBV}}^{2}}b\Delta G_{s}^{\prime }\\ \frac{\bar{i}}{\sigma_{I_{GEBV}}}SV_{GEBV_{\alpha L}} & 0 \end{array}\right]\left[\begin{array}{c} b\\ \eta \end{array}\right]=\left[\begin{array}{c} F^{\prime }\\ \Delta G_{s} \end{array}\right]$$

The inverse of the coefficient matrix of Eq. 4 can be obtained through inversion by partitioning [18]. As a result, index weights (*b*) of *I_GEBV_* can be obtained:


(5) $$b=\left\{I_{k}-\left[A-B\right]{\left(C\left[A-B\right]\right)}^{-1}C\right\}F^{\prime }+\left[A-B\right]{\left(C\left[A-B\right]\right)}^{-1}\;\Delta G_{s}$$

, where $A=\frac{\bar{i}}{\sigma_{I_{GESV}}}S^{\prime },\;B=\frac{1}{\sigma_{I_{GEBV}}^{2}}b\Delta G_{s}^{\prime }$, and $C=\frac{\bar{i}}{\sigma_{I_{GESV}}}SV_{GEBV_{\alpha L}}$.

Furthermore, the values of *b* can also be obtained directly by inversion of Eq. 4.

When the value of variance of the maximum growth index $\left(\sigma_{\sigma_{I_{GEBV}}}^{2}\right)$ is very large compared to the values of elements of the (k×s) matrix (*b*Δ*G'_s_*), the term of $\frac{1}{\sigma_{\sigma_{I_{GEBV}}}^{2}}b\Delta G_{s}^{\prime }(=B)$ in Eq. 4 can be neglected. In this situation,


(6) $$b=\left[I_{k}-S^{\prime }{\left(SV_{GEBV_{\alpha L}}S^{\prime }\right)}^{-1}SV_{GEBV_{\alpha L}}\right]F^{\prime }+\frac{\sigma_{I_{GEBV}}}{\bar{i}}S^{\prime }{\left(SV_{GEBV_{\alpha L}}S^{\prime }\right)}^{-1}\Delta G_{s}.$$

Eq. 4 has three distinctive characteristics: 1) the coefficient matrix of Eq. 4 is not symmetric and nonlinear (in terms of *b*); 2) the coefficient matrix contains the selection intensity (*ī*), indicating that index coefficients vary depending on the value of (*ī*) as specified before selection; and 3) the coefficient matrix contains the unknown solution *b*, thus requiring an iterative approach to solve the equations. To start the iteration, it is logical to set the unknown *b* to the unrestricted growth index weights. The unrestricted growth index (Supplement 1) maximizes growth throughout the fattening process without restrictions on weight gains at prespecified time points during growth process. Because Eqs. 5, 6 is not a function of *η*, there is no need to assume the initial values for *η*. In an animal breeding context, selection intensity (*ī*) and the elements of Δ*G_s_* are rather small constants. Eq. 4 has full rank (k+s). Therefore, iteration on Eq. 4 would converge to yield a unique solution. It is worth noting that the predetermined constrained weight gain should be biologically reasonable. It would be difficult to achieve restrictions when selection intensity is too small when the number of restrictions is large. A failure to achieve convergence in Eq. 4 is a good indication that the predetermined genetic gains at specific times during growth is more extreme than is genetically feasible and the selection intensity applied is too small. The iteration procedure is summarized as:

1) Prespecify *ī* and initial solutions for *b*;
2) Calculate $\sigma_{I_{GEBV}}=\sqrt{b^{\prime }V_{GEBV_{\alpha L}}b}$ based on *b* set in step 1);
3) Solve Eq. 4 for *b*. That is, *b* and *σ_I_*_*_GEBV_*_ are updated; and
4) Repeat steps 2) and 3) until convergence of *b*.

There are too many restrictions to satisfy the Eq. 3, i.e., $SV_{GEBV_{\alpha L}}b\frac{\bar{i}}{\sigma_{I_{GEBV}}}=\Delta G_{s}$, when the number of restrictions regarding desired gains (s) exceeds the number of RR coefficients fitted (k). In general, when difference in Legendre polynomial coefficients after and before selection is given (Δα*_L_*, i.e., α*_L_* after selection – α*_L_* before selection), an equation (*S*Δα*_L_ =* Δ*G_S_*) holds true without any restrictions on s and k, i.e., s>k, s<k, or s = k. On the other hand, the difference (Δα*_L_*) can only be obtained uniquely when the number of restrictions (s) is equal to the number of Legendre polynomial coefficients fitted to the growth function (k). Since $\Delta \alpha_{L}=V_{GEBV_{\alpha L}}b\frac{\bar{i}}{\sigma_{I_{GEBV}}}$ from Eq. 1, $S\Delta \alpha_{L}=SV_{GEBV_{\alpha L}}b\frac{\bar{i}}{\sigma_{I_{GEBV}}}=\Delta G_{s}$. The terms of Δα*_L_* and *b* are obtained uniquely when s = k. The term $\frac{\bar{i}}{\sigma_{I_{GEBV}}}$ in Eq. 3 must be 1 when s = k. Therefore, the term *σ_I_*_*_GEBV_*_ is a constant and cannot be differentiated with respect to *b*, even though *σ_I_*_*_GEBV_*_ is written as $\sqrt{b^{\prime }V_{GEBV_{\alpha L}}b}$. In addition, when the number of desired gains during growth is equal to the number of RR coefficients fitted (s = k), the RR covariate matrix *S* becomes an (s×s) square matrix and inverse of *S* is described as *S*^−1^. Therefore, *b* in Eq. 3 is described as:


(7) $$b={\left(V_{GEBV_{\alpha L}}\right)}^{-1}S^{-1}\Delta G_{s}$$

And selection intensity (*ī*) required to achieve Δ*G_s_* is $\sqrt{b^{\prime }V_{GEBV_{\alpha L}}b}$.

Eq. 3 is simply an equation that satisfies the intended weight gains at specified time points without regard to maximizing genetic gain throughout the growth process. That is, when the number of desired weight gains during growth is equal to the number of fitted RR coefficients, then the maximum growth index will be an index that only meets the desired weight gain at a particular time point, rather than maximizing growth during growth process. Therefore, the selection intensity cannot be specified in advance to a particular value.

Additionally, the previously reported index weights which satisfy the desired weight gains specified in advance while minimizing selection intensity was shown in [1,2], i.e.,


$$b=S^{\prime }{\left(SV_{GEBV_{\alpha L}}S^{\prime }\right)}^{-1}\;\Delta G_{s}.$$

Especially when s = k, *b* = *S*′(*SV_GEBV_*_*_αL_*_ *S*′)^−1^ Δ*G_s_* =


(8) $$S^{\prime }{\left(S^{\prime }\right)}^{-1}{\left(V_{GEBV_{\alpha L}}\right)}^{-1}S^{-1}\Delta G_{s}={\left(V_{GEBV_{\alpha L}}\right)}^{-1}S^{-1}\Delta G_{s}$$

Eq. 8 is the same as Eq. 7. In the end, when s = k, the index becomes a just valid index to satisfy the desired gains specified in advance without minimizing selection intensity or maximizing growth in whole growth process. Therefore, when s<k, while achieving the specified amount of gains during growth, the developed index in this study can maximize growth in whole growth process and the previously reported index [1,2] can minimize selection intensity. For example, in quartic equation (k = 5), the number of desired gains should be 4 or less to maximize growth during the entire growth process while achieving pre-determined desired gains at specific time points, because constant is included to the number of RR coefficients fitted (k).

### Numerical examples

Given that the purpose of this study was to develop an index rather than to apply Legendre RR to a growth curve, we assumed the birth weight; the body weights at 5, 81, 127, 128, and 130 weeks of age during the fattening process; the Legendre coefficients in the absence of selection (Supplement 2); and the genetic covariance matrix of Legendre polynomial coefficients (Supplement 3) in the same way as previously [1,2], which was based on the fattening process in Japanese Black steers [19,20].

The reliability of *GEBV* (*j = 0, 1, ... , k* − *1*) for the *j^th^* order of Legendre RR coefficients was assumed to be 0.7. In the current study, quartic Legendre polynomials were assumed as done previously [1,2]. Because Japanese Black steers are slaughtered at approximately 30 months of age [21], we fitted a growth curve to 130 weeks of age. We assumed that the growth curve in the absence of selection is similar to the curve derived from fitting a Gompertz growth curve [19]. Instead, we fitted a RR model for that curve to develop a selection index based on RR coefficients. With our selection index, the main goals of breeding are to have a smaller birth weight than at pre-selection and to reach the 130-week weight earlier. We set constraints on body weights in relation to the breeding goals and four examples of desired weight gains, as we did previously [2]. These examples reflect differences in how specific constraints on the amount of weight gain at specific times during growth are set. The four examples are shown in Table 1, that is,

Example 1: birth weight (−2.5 kg);

Example 2: birth weight (−2.5 kg) and weight at 128 weeks (+4.6 kg);

Example 3: birth weight (−2.5 kg), weight at 66 weeks (+2.8 kg), and weight at 128 weeks (+4.6 kg); and

Example 4: birth weight (−2.5 kg), weight at 43 weeks (+1.4 kg), weight at 87 weeks (+3.9 kg), and weight at 128 weeks (+4.6 kg).

The number of constraints in these examples (s = 1, 2, 3, or 4) is less than the number of quartic Legendre RR coefficients fitted including a constant (k = 5). Therefore, these constraints on desired body weight gains are achieved and the amount of growth is maximized throughout the growth process with the exception of the desired weight gain at pre-specified points.

## RESULTS AND DISCUSSION

### Index coefficients at convergence

Table 2 shows the index coefficients and total weight gains at convergence for selection intensities of 0.5 and 1.0 for constraint Examples 1, 2, 3, and 4. Convergence was reached earlier as the number of constraints on desired weight gains decreased. For example, when the intensity of selection was equal to 1.0, the number of rounds of iteration at convergence was 5, 6, 12, and 12 for 1, 2, 3, and 4 constraints, respectively. In addition, when the intensity of selection was 0.5, the number of rounds of iteration at convergence was 5, 9, 57, and 40 for 1, 2, 3, and 4 constraints, respectively. Therefore, convergence occurred later as the selection intensity decreased. The prespecified desired genetic weight gains (Δ*G_S_*) and the weight gains achieved with the maximum growth index were consistent for all the constraint Examples 1, 2, 3, and 4.

The total weight gain throughout growth (excluding gains at constrained weeks of age) decreased as the number of constraints on desired gains increased, indicating that the fewer the constraints, the more diverse the growth curve and the greater the potential for a greater total weight gain. Conversely, the growth curve is more controlled when the number of constraints increases. As a result, less constraint increases the likelihood of diverse growth curves and greater weight gain over the entire growth period. Even when the selection intensity increased from 0.5 to 1.0, total gain during growth (excluding gains at constrained weeks of age) did not double, because the intensity of selection is not independent of the index coefficients. The index coefficients vary depending on the specified selection intensity, even though the constraints on weight gain might be same; that is, different selection intensities result in different indices, even when the desired weight gain is the same.

### Comparison of weight gain achieved with the maximum growth index that maximizes total weight gain, the point-gain index that minimizes selection intensity, and an unrestricted growth index

We then compared the total weight gain throughout the growth process and weight gains at specific weeks of age between the maximum growth index, the point-gain index, and the unrestricted growth index. Table 3 shows the results for constraint Examples 1 and 2, and Table 4 shows the results for constraint Examples 3 and 4, with the specific conditions of the four Examples shown in Table 1. The numbers of constraint Examples 1, 2, 3, and 4 refer to the numbers of constraints on weight gain throughout the growth process. We applied the same selection intensity for all three indices; the value of the selection intensity was determined by using the point-gain index that achieved the desired weight gain at the lowest selection intensity. Overall, the selection intensity was relatively low, i.e., 0.0713, 0.1264, 0.4179, and 0.3965 for constraint Examples 1, 2, 3, and 4, respectively.

Desired gains were achieved for both the maximum growth index and point-gain indices. Although the unrestricted growth index yielded the highest total gain among the three indices compared, this index failed to achieve the desired gains, because it aims only to achieve the maximum weight gain throughout the growth process. Total weight gain throughout the growth phase was negative for constraint Example 1, where only birth weight was constrained (−2.5 kg), and it was −103.6 and −127.0 for the maximum growth index and point-gain index, respectively. The maximum growth index maximized the total weight gain throughout the growth process and achieved the desired weight gains at specified time points. However, the total weight gain was negative even for the maximum growth index (Table 3), because the selection intensity was 0.0713 and therefore almost zero. In contrast, with the unrestricted growth index, which maximizes weight gain throughout the entire growth period without the restriction of reducing the birth weight by 2.5 kg, the birth weight increased by 0.88 kg, and the overall weight gain during growth was 369 kg even though the selection intensity was only 0.0713.

In constraint Examples 2, 3, and 4, the total weight gain was greater for the maximum growth index than for the point-gain index. However, when the number of constraints was 3 or 4, this tendency was less evident. As mentioned earlier, this is because the growth curve is less diverse and more controlled with a large number of constraints than with a small number of constraints.

### Genetic gain from birth to the end of fattening, daily gain, and body weight after selection

**Figure 1:**
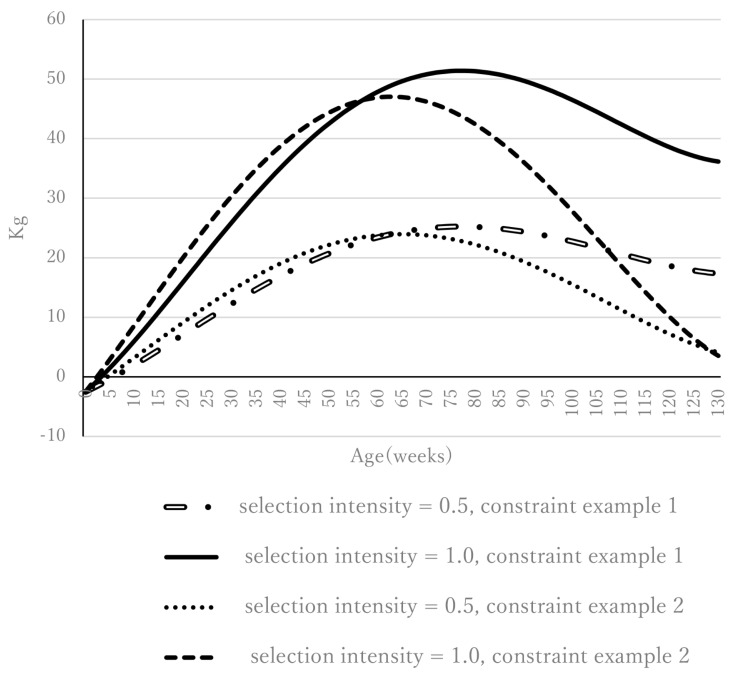
Genetic gains from the maximum growth index for constraint examples 1 and 2 for selection intensities of 0.5 and 1.0.

Genetic gains from the maximum growth index for constraint Examples 1 and 2 are shown for selection intensities of 0.5 and 1.0 (Figure 1). For constraint Example 1 (constraint on birth weight only [−2.5 kg]), for selection intensities 0.5 and 1.0, genetic gain increased with age toward 77 weeks and decreased gradually thereafter to the end of fattening. For constraint Example 2 (i.e., constraints on weight at birth [−2.5 kg] and 128 weeks [4.6 kg]), genetic gain increased with age toward 65 and 63 weeks for selection intensities of 0.5 and 1.0, respectively. After it peaked, genetic gain decreased toward the end of fattening, and the decreasing trend was steeper in constraint Example 2 than in Example 1. This difference occurred because genetic gain at 128 weeks was constrained to 4.6 kg in constraint Example 2, whereas constraint Example 1 has no restriction on genetic gain other than the decrease at birth. Although genetic gain was greater at a selection intensity of 1.0 than at 0.5, the trend in genetic gain along the growth trajectory was not parallel between these selection intensities. As mentioned earlier, this lack of parallelism is because the selection index is different for each selection intensity. The genetic gains for constraint Example 3 (constraints on birth weight [−2.5 kg], weight at 66 weeks [20.2 kg], and weight at 128 weeks [4.6 kg]) derived from the maximum growth index for selection intensities of 0.5 and 1.0 are shown in Supplement 4. Similarly, the genetic gains for constraint Example 4 (constraints on birth weight [−2.5 kg], weight at 43 weeks [15.7 kg], weight at 87 weeks [16.6 kg] and weight at 128 weeks [4.6 kg]) derived from the maximum growth index for selection intensities of 0.5 and 1.0 are shown in Supplement 5. The number of constraints on weight gain during growth is 3 and 4 in Supplements 4, 5, respectively. Weight gain between the constrained ages showed an upward convex curve because the amount of increase due to the upward convex curve is larger than the linear increase. In other words, the index that we developed maximizes the amount of weight gain over the entire growth period, so that the amount of weight gain between the constrained ages creates an upward convex curve.

As mentioned earlier, genetic weight gain at age t can be described as a linear combination of the Legendre polynomial at age t standardized and the difference in Legendre RR coefficients between after and before selection. This can be written in matrix form as *S*Δ*α_L_* = Δ*G_S_*. Similarly, an index to achieve the intended differences in the Gompertz growth curve can be constructed by using the intended differences in the parameters A, B, and K, which are asymptotic weight, growth starting point, and maturity rate, respectively (Supplement 6). However, the genetic weight gain at age t cannot be expressed as a linear combination of the polynomial evaluated at age t and the differences in the parameters of the Gompertz growth curve between after and before selection. Therefore, using the RR growth curve and expressing the genetic weight gain at age t as a linear combination of the polynomial evaluated at age t and the differences in the parameters of the Legendre growth curve between after and before selection made it possible to maximize the amount of genetic gain throughout the growth process and concurrently achieve the desirable genetic gain at a particular growth point.

**Figure 2:**
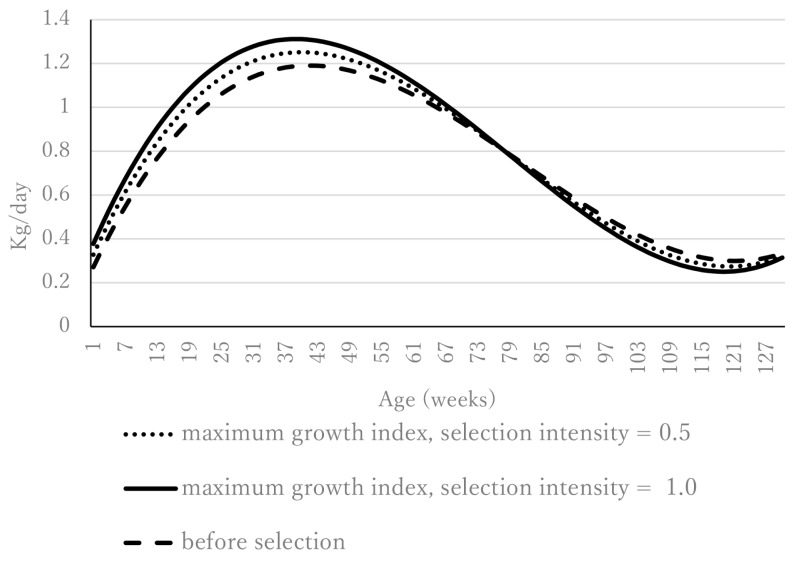
Daily gain from the maximum growth index (the only constraint is birth weight [−2.5 kg]).

**Figure 3:**
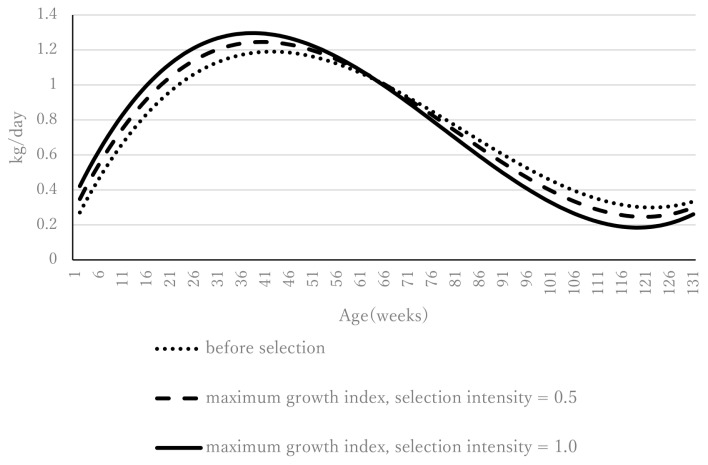
Daily gains from the maximum growth index for selection intensities of 0.5 and 1.0 (constraints are birth weight [−2.5 kg] and weight at 128 weeks [4.6 kg]).

The daily gain based on the maximum growth index constrained only by birth weight (−2.5 kg) is shown in Figure 2. Before selection, daily gain peaked at 41 weeks of age (1.19 kg). For the maximum growth index at a selection intensity of 1.0, daily gain peaked at 39 weeks of age—earlier than before selection—and the daily gain at the peak was 1.31 kg, which was greater than before selection. This tendency was more pronounced at a selection intensity of 1.0 than at 0.5. The daily gain from the maximum growth index at selection intensities 0.5 and 1.0 with constraints on birth weight (−2.5 kg) and 128-week weight (4.6 kg) is shown in Figure 3. In the same way as in Figure 2, where constraints were set on birth weight only, daily gain peaked earlier than before selection, and the daily gain at peak was greater than before selection. For example, under a selection intensity of 1.0, the maximum daily gain (+1.30 kg) occurred at 37 weeks of age, compared with a maximum daily gain of +1.19 kg at 41 weeks of age before selection. Furthermore, the rate of decline in daily gain after the peak was steeper after selection than before.

Daily gain from the maximum growth index for constraint Examples 3 and 4 at a selection intensity of 1.0 is shown in Supplement 7. In the same way as for Figures 2, 3, maximum daily gain peaked earlier and higher with the maximum growth index selection than before selection. Daily gain after the peak decreased more steeply after selection than before selection. For the maximum growth index as shown in Figures 2, 3 and Supplement 7, birth weight was constrained to decrease by 2.5 kg. Given that the maximum average daily gain in late-maturing pigs peaked 10 days later than in early maturing pigs [22], an earlier growth peak after selection than before appears to be associated with precocious maturation. A maximum growth index in which birth weight is constrained to decrease might lead to transition to a precocious growth curve.

As mentioned earlier, to achieve the desired weight gain, the maximum number of constraints on weight at given time points must be less than the number of RR coefficients fitted including a constant (s<k). Therefore, the maximum growth index we developed can be easily applied in breeding practice because, in the case of a single constraint, such as reduced birth weight, any Legendre RR, whether quadratic, cubic, or quartic, can maximize weight gain during growth.

**Figure 4:**
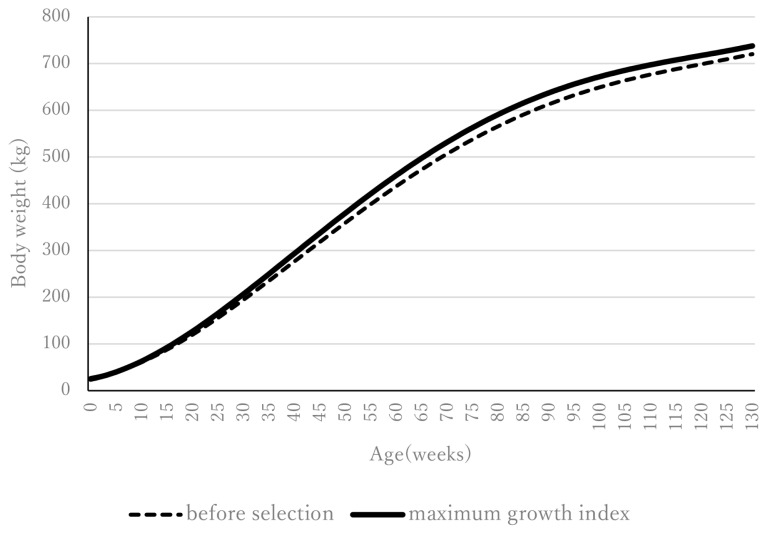
Body weight from the maximum growth index for selection intensity 0.5 (the only constraint is birth weight [−2.5 kg]).

By developing a maximum growth index that suppresses birth weight by 2.5 kg and maximizes growth during the fattening period, we achieved a lower birth weight and heavier final weight than before selection (Figure 4); that is, the index we developed makes it possible to reach the pre-selection final weight 8 weeks earlier and to increase the post-selection final weight by 17 kg compared with that at pre-selection (737.7 kg vs. 720.4 kg), even when selection intensity is set to 0.5 only. At a selection intensity of 0.5, approximately 70% of the population is selected, and only 30% is culled, therefore suggesting weak selection. Increase in inbreeding is not that serious at a selection intensity of 0.5. Therefore, even at a low selection intensity of 0.5, the maximum growth index enables a large amount of growth. The property that the maximum growth index maximizes growth over the entire growth period reduces the need for high selection intensity required to achieve large amounts of weight gain. However, what is noteworthy is the selection intensity in the previously reported point-gain index was 0.127 when the number of constraints was two (i.e., constraints on weight at birth [−2.5 kg] and 128 weeks [4.6 kg]) [1]. That selection intensity of 0.127 was very low. Even at a very low selection intensity, the previously reported point-gain index made it possible to achieve desirable weight gains at birth and at 128 weeks, with a total weight gain throughout the growth period reaching 223 kg [1]. In addition, the lower the selection intensity, the more likely the selection goal will be achieved. That characteristics of the point-gain index helps maintain genetic diversity. Therefore, when low selection intensity is prioritized from a long-term perspective of genetic diversity, a point-gain index is also necessary. Which method to use—suppressing inbreeding or maximizing overall growth—is the breeder’s decision, considering the characteristics of the maximum growth index and the point-gain index. The selection index procedure that we developed might easily be applied to other longitudinal data such as growth curves in plants and egg-production curves in poultry. Although this study uses GEBVs of the RR coefficients, EBV or phenotype itself [2] of the RR coefficients can be treated as selection index traits of the maximum growth index. EBV coefficients can be applied simply substituting GEBV for EBV in EBV maximum growth index. Phenotypic maximum growth index is written in Supplement 8.

## CONCLUSION

We developed a selection index for maximizing total weight gain throughout growth in beef cattle, while constraining birth weight and other traits. The maximum growth index developed in this study enabled a reduction in birth weight despite an increased final body weight. Moderating the birth weight of calves leads to fewer dystocia. The index that we developed will help to solve the problem that increased final weight leads to increased birth weight because of the positive genetic correlation between final weight and birth weight. The RR coefficients can be treated as selection index traits of the maximum growth index. Although this study used GEBVs of the RR coefficients, EBV and phenotypic maximum growth index was briefly explained.

## Figures and Tables

**Table 1:** Constraint examples 1 through 4

Example					
1	Constrained week	0			
	Constrained body weight gain (kg)	−2.5			
2	Constrained week	0	128		
	Constrained body weight gain (kg)	−2.5	4.6		
3	Constrained week	0	66	128	
	Constrained body weight gain (kg)	−2.5	20.2	4.6	
4	Constrained week	0	43	87	128
	Constrained body weight gain (kg)	−2.5	15.7	16.6	4.6

**Table 2:** Numbers of rounds of iteration and index coefficients at convergence for the four constraint examples when the given weight gains are reached and the selection intensity is 0.5 or 1.0

	Constraint example^1)^			

	1	2	3	4
Selection intensity = 0.5				
Number of rounds at convergence	5	9	57	40
Index coefficient for constant	41.874	27.106	7.440	3.004
Index coefficient for 1st order	87.910	0.093	−0.002	0.052
Index coefficient for 2nd order	−111.904	−138.168	−28.730	−11.264
Index coefficient for 3rd order	134.295	13.056	12.209	4.329
Index coefficient for 4th order	−150.094	−165.781	−149.346	−68.587
Total gain during growth excluding gains at constrained weeks of age (kg)	2,277.4	1,924.4	1,694.5	1,557.0
Selection intensity = 1.0				
Number of rounds at convergence	5	6	12	12
Index coefficient for constant	48.988	29.700	5.161	2.141
Index coefficient for 1st order	75.588	−21.559	−1.089	−0.382
Index coefficient for 2nd order	−95.996	−131.346	−14.995	−6.179
Index coefficient for 3rd order	115.471	−18.066	10.559	3.944
Index coefficient for 4th order	−128.751	−153.670	−146.732	−64.573
Total gain during growth excluding gains at constrained weeks of age (kg)	4,719.1	3,718.5	1,923.3	1,652.3

1) Constraint examples are explained in Table 1.

**Table 3:** Total weight gain (kg) throughout the growth process and weight gains (kg) at specific weeks of age among the maximum growth index, point-gain index, and unrestricted growth index when the constraint examples were 1 and 2

Constraint example^1)^						

Weeks of age	1			2		

	Gain at birth constrained to −2.5kg			Gains at birth and at 128 weeks of age constrained to −2.5 and 4.6 kg, respectively		

	Selection intensity = 0.0713^2)^			Selection intensity = 0.1264		

	Maximum growth index^3)^	Point-gain index^4)^	Unrestricted growth index^5)^	Maximum growth index	Point-gain index	Unrestricted growth index
0	−2.50	−2.50	0.88	−2.50	−2.50	1.55
21	−1.41	−1.50	1.76	−0.98	−1.17	3.11
43	−0.68	−0.87	2.87	0.84	0.50	5.09
66	−0.33	−0.57	3.58	2.51	2.14	6.34
87	−0.31	−0.55	3.60	3.61	3.32	6.39
128	−0.94	−1.12	2.91	4.60	4.60	5.15
130	−0.98	−1.16	2.91	4.62	4.64	5.15
Total gain (kg)^6)^	−103.62	−127.03	369.00	245.81	216.57	653.85

1) Constraint examples are explained in Table 1.

2) Selection intensity was set to the magnitude of the point-gain index that minimizes selection intensity.

3) Maximum growth index maximizes growth during growth while constraining the prespecified weight gains.

4) Point-gain index achieves the prespecified gains while minimizing selection intensity.

5) Unrestricted growth index maximizes growth during the entire growth process without achieving prespecified gains.

6) Total gain refers to the gain during the entire growth process, excluding gains at constrained weeks of age.

**Table 4:** Total weight gain (kg) throughout the growth process and weight gains (kg) at specific weeks of age among the maximum growth index, point-gain index, and unrestricted growth index when the constraint examples were 3 and 4

Constraint example^1)^						

Age (weeks)	3			4		

	Gains at birth and at 66 and 128 weeks of age were constrained to −2.5, 20.2, and 4.6 kg, respectively			Gains at birth and at 43, 87, and 128 weeks of age were constrained to −2.5, 15.7, 16.6, and 4.6 kg, respectively		

	Selection intensity = 0.4179^2)^			Selection intensity = 0.3965		

	Maximum growth index^3)^	Point-gain index^4)^	Unrestricted growth index^5)^	Maximum growth index	Point-gain index	Unrestricted growth index
0	−2.50	−2.50	5.13	−2.50	−2.50	4.87
21	7.71	7.57	10.29	7.19	7.14	9.77
43	16.66	16.63	16.83	15.70	15.70	15.97
66	20.16	20.20	20.97	19.11	19.15	19.90
87	17.35	17.33	21.13	16.59	16.60	20.05
128	4.60	4.60	17.03	4.60	4.60	16.16
130	4.20	4.25	17.03	4.22	4.23	16.16
Total gain (kg)^6)^	1,601.86	1,595.35	2,124.70	1,510.83	1,509.66	2,015.95

1) Constraint examples are explained in Table 1.

2) Selection intensity was set to the magnitude of the point-gain index that minimizes selection intensity.

3) Maximum growth index maximizes growth throughout the growth process yet constrains the prespecified weight gains.

4) Point-gain index achieves the prespecified gains and minimizes selection intensity.

5) Unrestricted growth index maximizes growth throughout the entire growth process without achieving prespecified gains.

6) Total gain refers to the gain throughout the entire growth process, excluding gains at constrained weeks of age.

## SUPPLEMENTARY MATERIAL

**Supplement 1.** Unrestricted growth index (*I**_u_*).

**Supplement 2.** Birth weight; body weight at various ages; and Legendre coefficients before selection.

**Supplement 3.** Genetic (co)variances of Legendre coefficients (kg).

**Supplement 4.** Genetic gains from the maximum growth index for selection intensities of 0.5 and 1.0 (constraints on birth weight [−2.5kg], weight at 66 weeks [20.2 kg], and weight at 128 weeks [4.6 kg]).

**Supplement 5.** Genetic gains from the maximum growth index for selection intensities of 0.5 and 1.0, with constraints on birth weight [−2.5 kg], weight at 43 weeks [15.7 kg], weight at 87 weeks [16.6 kg], and weight at 128 weeks [4.6 kg].

**Supplement 6.** Gompertz growth curve.

**Supplement 7.** Daily gains from the maximum growth index for constraint examples 3 and 4 when the selection intensity is 1.0.

**Supplement 8.** Phenotypic maximum growth index.

## References

[1] Togashi K Watanabe T Ogino A Development of an index that decreases birth weight, promotes postnatal growth and yet minimizes selection intensity in beef cattle Anim Biosci 2024 37 839 51 10.5713/ab.23.0343 38271985 PMC11065704

[2] Togashi K Ogino A Watanabe T Genomic and phenotypic selection indices for decreased birth weight, shortening fattening period, and regulating total weight gain in beef cattle Anim Biosci 2025 38 223 35 10.5713/ab.24.0171 39210817 PMC11725746

[3] Colburn DJ Deutscher GH Nielsen MK Adams DC Effects of sire, dam traits, calf traits, and environment on dystocia and subsequent reproduction of two-year-old heifers J Anim Sci 1997 75 1452 60 10.2527/1997.7561452x 9250504

[4] Nix JM Spitzer JC Grimes LW Burns GL Plyler BB A retrospective analysis of factors contributing to calf mortality and dystocia in beef cattle Theriogenology 1998 49 1515 23 10.1016/s0093-691x(98)00097-1 10732015

[5] Hickson RE Morris ST Kenyon PR Lopez-Villalobos N Dystocia in beef heifers: a review of genetic and nutritional influences N Z Vet J 2006 54 256 64 10.1080/00480169.2006.36708 17151722

[6] Fitzhugh HA Jr Analysis of growth curves and strategies for altering their shape J Anim Sci 1976 42 1036 51 10.2527/jas1976.4241036x 770411

[7] Schaeffer LR Random regressions in animal models for test-day production in dairy cattle Proceedings of the 5th World Congress of Genetics Applied Livestock Production 1994 Aug 11 Guelph, ON University of Guelph 1994

[8] Jamrozik J Schaeffer LR Dekkers JCM Genetic evaluation of dairy cattle using test day yields and random regression model J Dairy Sci 1997 80 1217 26 10.3168/jds.S0022-0302(97)76050-8 9201594

[9] Togashi K Lin CY Modifying the lactation curve to improve lactation milk and persistency J Dairy Sci 2003 86 1487 93 10.3168/jds.S0022-0302(03)73734-5 12741575

[10] Togashi K Lin CY Development of an optimal index to improve lactation yield and persistency with the least selection intensity J Dairy Sci 2004 87 3047 52 10.3168/jds.S0022-0302(04)73437-2 15375067

[11] Boligon AA Mercadante MEZ Forni S Lôbo RB Albuquerque LG Covariance functions for body weight from birth to maturity in Nellore cows J Anim Sci 2010 88 849 59 10.2527/jas.2008-1511 19897625

[12] Meyer K Scope for a random regression model in genetic evaluation of beef cattle for growth Livest Prod Sci 2004 86 69 83 10.1016/S0301-6226(03)00142-8

[13] Meyer K Random regression analyses using B-splines to model growth of Australian Angus cattle Genet Sel Evol 2005 37 473 500 10.1186/1297-9686-37-6-473 16093011 PMC2697221

[14] Mota LFM Martins PGMA Littiere TO Abreu LRA Silva MA Bonafé CM Genetic evaluation and selection response for growth in meat-type quail through random regression models using B-spline functions and Legendre polynomials animal 2018 12 667 74 10.1017/S1751731117001951 28803586

[15] Přibyl J Krejčová H Přibylová J Misztal I Bohmanová J Štípková M Trajectory of body weight of performance tested dual-purpose bulls Czech J Anim Sci 2007 52 315 24 10.17221/2340-CJAS

[16] Oliveira HR Brito LF Lourenco DAL Invited review: advances and applications of random regression models: from quantitative genetics to genomics J Dairy Sci 2019 102 7664 83 10.3168/jds.2019-16265 31255270

[17] Henderson CR Applications of linear models in animal breeding University of Guelph 1984

[18] Searle SR Matrix Algebra for the biological sciences John Wiley & Sons 1966

[19] Takeda M Uemoto Y Inoue K Evaluation of feed efficiency traits for genetic improvement in Japanese Black cattle J Anim Sci 2018 96 797 805 10.1093/jas/skx054 29584931 PMC6093584

[20] Onogi A Ogino A Sato A Kurogi K Yasumori T Togashi K Development of a structural growth curve model that considers the causal effect of initial phenotypes Genet Sel Evol 2019 51 1 9 10.1186/s12711-019-0461-y 31046678 PMC6498631

[21] National Agricultural Research Organization Japanese feeding standard beef cattle [Internet] Japan livestock Industry Association c2008 [cited 2024 Jun 21]. Available from: https://www.naro.go.jp/laboratory/nilgs/contents/shiryo_hyojyun/nikuyou2008/index.html

[22] Faccin J Early vs. late maturing sire lines [Internet] Pigchamp c2023 [cited 2024 Mar 11]. Available from: https://www.pigchamp.com/news/benchmark-magazine/articles/ArtMID/2128/ArticleID/307/Early-vs-Late-Maturing-Sire-Lines

