# Supplementary material for: Selection index for beef cattle that maximizes overall growth yet constraining birth weight and other traits
Source: Anim Biosci. 2025 Aug 12;39(1):240912. doi: 10.5713/ab.24.0912 (PMC12754505; doi:10.5713/ab.24.0912)
Supplement: Supplementary file 1 [file ab-24-0912-Supplementary-1.pdf]

Supplement 1. Unrestricted growth index (I_u)

The unrestricted growth index (I_u) aims to maximize the weight gain throughout growth (G_L) without restrictions on weight gain at specific time points.

Given that

$I_u = \boldsymbol{b_u}'\boldsymbol{GEBV_{\alpha L}}$ and that $\boldsymbol{b_u}$ is a column vector of index weights of unrestricted growth index (I_u), the correlation (r_{I_u,G_L}) between I_u and G_L can be shown as

$$r_{I_u,G_L} = \frac{\boldsymbol{b_u}'\boldsymbol{V_{GEBV_{\alpha L}}}\boldsymbol{F}'}{\sqrt{\boldsymbol{b_u}'\boldsymbol{V_{GEBV_{\alpha L}}}\boldsymbol{b_u}}\sqrt{\boldsymbol{FKF}'}} .$$

Differentiating the function r_{I_u,G_L} with respect to $\boldsymbol{b_u}$ and equating the resulting partial derivatives to zeros results in the following equation:

$$\frac{1}{\sqrt{\boldsymbol{FKF}'}}\frac{1}{\boldsymbol{b_u}'\boldsymbol{V_{GEBV_{\alpha L}}}\boldsymbol{b_u}}\left[\sqrt{\boldsymbol{b_u}'\boldsymbol{V_{GEBV_{\alpha L}}}\boldsymbol{b_u}}\boldsymbol{V_{GEBV_{\alpha L}}}\boldsymbol{F}' - \boldsymbol{b_u}'\boldsymbol{V_{GEBV_{\alpha L}}}\boldsymbol{F}'\frac{\boldsymbol{V_{GEBV_{\alpha L}}}\boldsymbol{b_u}}{\sqrt{\boldsymbol{b_u}'\boldsymbol{V_{GEBV_{\alpha L}}}\boldsymbol{b_u}}}\right] = \boldsymbol{0}.$$

Transforming this equation leads to:

$$\boldsymbol{V_{GEBV_{\alpha L}}}\boldsymbol{b_u} = \boldsymbol{V_{GEBV_{\alpha L}}}\boldsymbol{F}'\frac{\boldsymbol{b_u}'\boldsymbol{V_{GEBV_{\alpha L}}}\boldsymbol{b_u}}{\boldsymbol{b_u}'\boldsymbol{V_{GEBV_{\alpha L}}}\boldsymbol{F}'}$$

The scalar $\frac{\boldsymbol{b_u}'\boldsymbol{V_{GEBV_{\alpha L}}}\boldsymbol{b_u}}{\boldsymbol{b_u}'\boldsymbol{V_{GEBV_{\alpha L}}}\boldsymbol{F}'}$ can be omitted without affecting the proportionality of $\boldsymbol{b_u}$, such that

$\boldsymbol{V_{GEBV_{\alpha L}}}\boldsymbol{b_u} = \boldsymbol{V_{GEBV_{\alpha L}}}\boldsymbol{F}'$ and $\boldsymbol{b_u} = \boldsymbol{F}'$.

In addition, the genetic gain at i[th] time during growth (ΔG_{ti}) can be described as:

$$\Delta G_{ti} = cov(\boldsymbol{s_i}\boldsymbol{\alpha_L}, I'_u)\frac{\bar{\iota}}{\sigma_{I_u}^2}\sigma_{I_u} = \boldsymbol{s_i}\boldsymbol{V_{GEBV_{\alpha L}}}\boldsymbol{F}'\frac{\bar{\iota}}{\sigma_{I_u}} , \qquad (1)$$

where $\boldsymbol{s_i} = [\varphi_0(t_i) \quad \varphi_1(t_i) \quad \varphi_2(t_i) \quad .. \quad \varphi_{k-1}(t_i)]$ and $\varphi_{\boldsymbol{j}}(t_i)$ is the jth order of

Legendre polynomial ($j=0,..,k-1$) evaluated at age t_i standardized.

Note that the selection intensity in Eq. (1) is independent of the index coefficients of unrestricted growth index and that any magnitude of intensity can be used, resulting in a genetic gain that is proportional to the intensity of selection.
