# Supplementary material for: Selection index for beef cattle that maximizes overall growth yet constraining birth weight and other traits
Source: Anim Biosci. 2025 Aug 12;39(1):240912. doi: 10.5713/ab.24.0912 (PMC12754505; doi:10.5713/ab.24.0912)
Supplement: Supplementary file 2 [file ab-24-0912-Supplementary-2.pdf]

Supplement 2. Birth weight; body weight at various ages; and Legendre coefficients before selection

Birth weight (kg)	27.4
Body weight (kg) at	
5 weeks	40.2
81 weeks	570.7
127 weeks	713.5
128 weeks	715.8
130 weeks	720.4
Legendre coefficient	
$\alpha 0$[1)]	601.4
$\alpha 1$	317.9
$\alpha 2$	–48.3
$\alpha 3$	–22.9
$\alpha 4$	11.8

[1)] αi indicates order i.
