# Supplementary material for: Selection index for beef cattle that maximizes overall growth yet constraining birth weight and other traits
Source: Anim Biosci. 2025 Aug 12;39(1):240912. doi: 10.5713/ab.24.0912 (PMC12754505; doi:10.5713/ab.24.0912)
Supplement: Supplementary file 3 [file ab-24-0912-Supplementary-3.pdf]

Supplement 3. Genetic (co)variances of Legendre coefficients (kg)

Order	0	1	2	3	4	CV[1)]
0	4502.4	1367.7	–1270.1	–25.2	227.1	0.112
1		1062.8	67.7	–154.6	11.2	0.103
2			698.2	–88.6	–110.5	0.547
3		symmetric		38.1	10.0	0.270
4					18.3	0.363

[1)] Coefficient of variation = square root of genetic variance of Legendre coefficient / Legendre coefficient before selection (see Supplement 2).
