# Supplementary figures and images for: Selection index for beef cattle that maximizes overall growth yet constraining birth weight and other traits
Source: Anim Biosci. 2025 Aug 12;39(1):240912. doi: 10.5713/ab.24.0912 (PMC12754505; doi:10.5713/ab.24.0912)

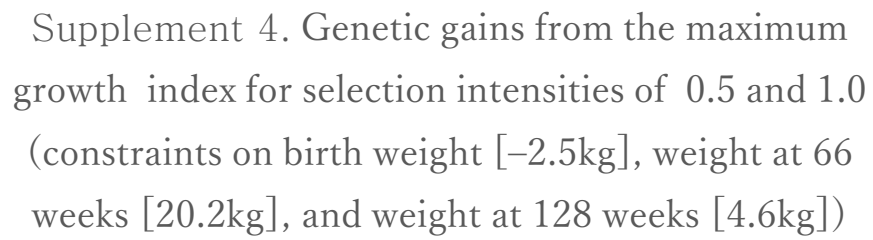

Supplement: Supplementary file 4 [file ab-24-0912-Supplementary-4.pdf]
