# Supplementary material for: Selection index for beef cattle that maximizes overall growth yet constraining birth weight and other traits
Source: Anim Biosci. 2025 Aug 12;39(1):240912. doi: 10.5713/ab.24.0912 (PMC12754505; doi:10.5713/ab.24.0912)
Supplement: Supplementary file 5 [file ab-24-0912-Supplementary-5.pdf]

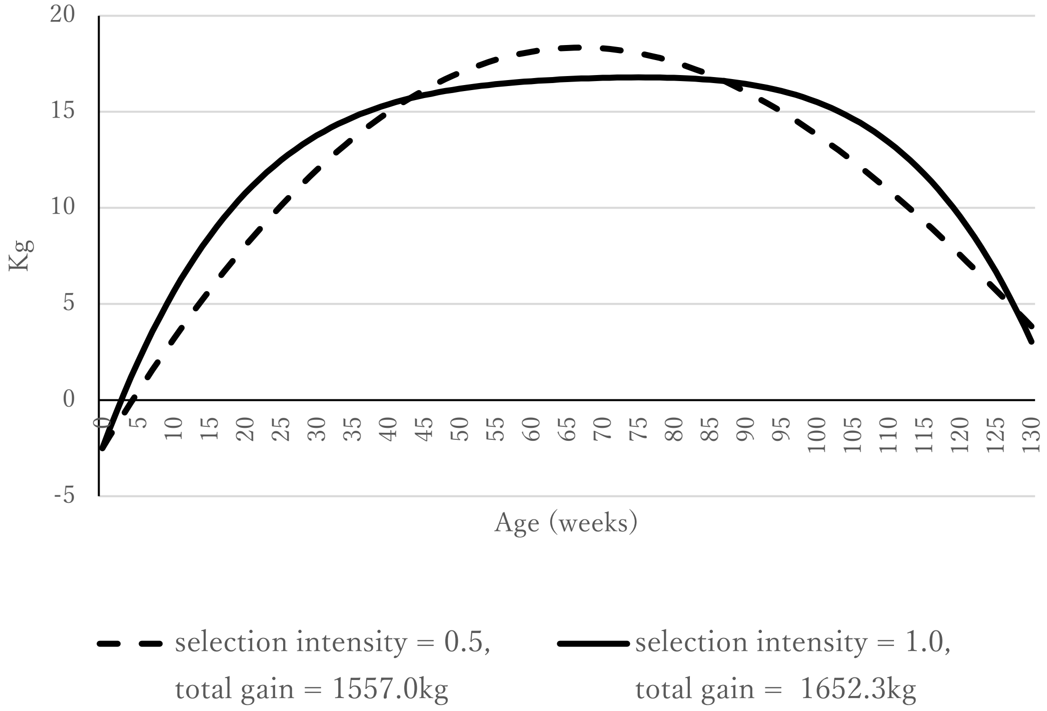

Supplement 5. Genetic gains from the maximum growth index for selection intensities of 0.5 and 1.0, with constraints on birth weight [- 2.5kg], weight at 43 weeks [15.7kg], weight at 87 weeks [16.6kg], and weight at 128 weeks [4.6kg]
