# Supplementary material for: Selection index for beef cattle that maximizes overall growth yet constraining birth weight and other traits
Source: Anim Biosci. 2025 Aug 12;39(1):240912. doi: 10.5713/ab.24.0912 (PMC12754505; doi:10.5713/ab.24.0912)
Supplement: Supplementary file 6 [file ab-24-0912-Supplementary-6.pdf]

Supplement 6. Gompertz growth curve

The Gompertz growth curve is expressed as

$y = Ae^{-Be^{-Kt}}$,

where A, B, and K are parameters for asymptotic weight, growth starting point, and maturity rate, respectively. An index that treats the parameters of the Gompertz curve as index traits aims to achieve breeders' desired differences in these parameters, as stipulated before selection. The index $(I_G = b_1A + b_2B + b_3K)$ to achieve these desired differences can be described as:

$$\begin{bmatrix}\Delta A\\ \Delta B\\ \Delta K\end{bmatrix} = cov\left(\begin{bmatrix}A\\ B\\ K\end{bmatrix}, [A \quad B \quad K]\right)\begin{bmatrix}b_1\\ b_2\\ b_3\end{bmatrix}\frac{\bar{\iota}}{\sigma_{I_G}}$$

$$= \begin{bmatrix}\sigma_A^2 & \sigma_{AB} & \sigma_{AK}\\ \sigma_{AB} & \sigma_B^2 & \sigma_{BK}\\ \sigma_{AK} & \sigma_{BK} & \sigma_K^2\end{bmatrix}\begin{bmatrix}b_1\\ b_2\\ b_3\end{bmatrix}\frac{\bar{\iota}}{\sigma_{I_G}},$$

where ΔA, ΔB, and ΔK are the differences in the parameters and $\bar{\iota}$ is the selection intensity. The selection intensity ($\bar{\iota}$) required to achieve $\begin{bmatrix}\Delta A\\ \Delta B\\ \Delta K\end{bmatrix}$ can be obtained by setting $\bar{\iota} = \sigma_{I_G}$. Therefore, the solution to the index weights ($b_1, b_2,$ and b_3) becomes:

$$\begin{bmatrix}b_1\\ b_2\\ b_3\end{bmatrix} = \begin{bmatrix}\sigma_A^2 & \sigma_{AB} & \sigma_{AK}\\ \sigma_{AB} & \sigma_B^2 & \sigma_{BK}\\ \sigma_{AK} & \sigma_{BK} & \sigma_K^2\end{bmatrix}^{-1}\begin{bmatrix}\Delta A\\ \Delta B\\ \Delta K\end{bmatrix}.$$

$$V(I_G) = \sigma_{I_G}^2 = [b_1 \quad b_2 \quad b_3]\begin{bmatrix}\sigma_A^2 & \sigma_{AB} & \sigma_{AK}\\ \sigma_{AB} & \sigma_B^2 & \sigma_{BK}\\ \sigma_{AK} & \sigma_{BK} & \sigma_K^2\end{bmatrix}\begin{bmatrix}b_1\\ b_2\\ b_3\end{bmatrix} = [b_1 \quad b_2 \quad b_3]\begin{bmatrix}\Delta A\\ \Delta B\\ \Delta K\end{bmatrix}.$$

Selection intensity ($\bar{\iota}$) is obtained as σ_{I_G}.
