# Supplementary material for: Selection index for beef cattle that maximizes overall growth yet constraining birth weight and other traits
Source: Anim Biosci. 2025 Aug 12;39(1):240912. doi: 10.5713/ab.24.0912 (PMC12754505; doi:10.5713/ab.24.0912)
Supplement: Supplementary file 7 [file ab-24-0912-Supplementary-7.pdf]

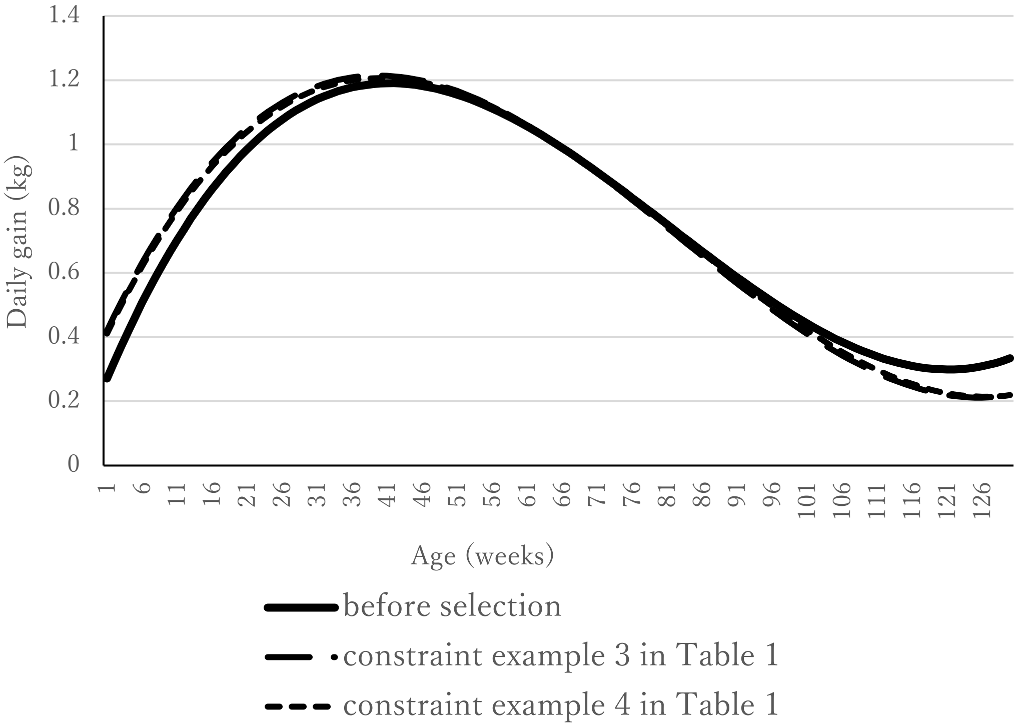

Supplement 7. Daily gains from the maximum growth index for constraint examples 3 and 4 when selection intensity is 1.0
