# Supplementary material for: Selection index for beef cattle that maximizes overall growth yet constraining birth weight and other traits
Source: Anim Biosci. 2025 Aug 12;39(1):240912. doi: 10.5713/ab.24.0912 (PMC12754505; doi:10.5713/ab.24.0912)
Supplement: Supplementary file 8 [file ab-24-0912-Supplementary-8.pdf]

Supplement 8. Phenotypic maximum growth index

1. **Phenotypic maximum growth index ($I_{\hat{P}}$) based on the average phenotypic RR growth curve coefficients of n progeny of a candidate bull**

$I_{\hat{P}} = \sum_{j=0}^{k-1} b_j \hat{P}_j = \boldsymbol{b}\hat{\boldsymbol{P}}$,

where $\hat{P}_j$ is the average phenotypic RR growth curve coefficients of n progeny of a candidate bull. Index weights ($\boldsymbol{b}$) can be obtained by iteration based on inversion the following matrix (1).

$$\begin{bmatrix} \boldsymbol{P_{\alpha L}} & \bar{\iota}\sqrt{\boldsymbol{b'P_{\alpha L}b}}\boldsymbol{G_{\alpha L}S'} - \boldsymbol{P_{\alpha L}b}\Delta\boldsymbol{G'}_s \\ \frac{\bar{\iota}}{\sqrt{\boldsymbol{b'P_{\alpha L}\,b}}}\boldsymbol{SG_{\alpha L}} & \boldsymbol{0} \end{bmatrix} \begin{bmatrix} \boldsymbol{b} \\ \boldsymbol{\eta} \end{bmatrix} = \begin{bmatrix} \boldsymbol{G_{\alpha L}F'} \\ \Delta\boldsymbol{G_s} \end{bmatrix} \quad (1)$$

, where $\boldsymbol{P_{\alpha L}}$ is a (k× k) phenotypic (co)variance matrix of $\hat{P}_j$, k is the number of Legendre coefficients, $\bar{\iota}$ is selection intensity, $\boldsymbol{G_{\alpha L}}$ is a (k× k) genetic (co)variance matrix for the RR growth curve coefficients, $\Delta\boldsymbol{G_s}$ is an (s × 1) column vector of the s desired weight gains, $\boldsymbol{S}$ is an (s × k) matrix as shown in Materials and Methods, $\boldsymbol{\eta}$ is an (s × 1) vector of Lagrange multipliers, and $\boldsymbol{F} = \sum_{i=1}^{m} \boldsymbol{F}_i$, $\boldsymbol{F}$ is a (1 × k) row vector, $F_{i,j}$ is the jth Legendre polynomial of covariate in the ith specific time.

In the element of $\boldsymbol{P_{\alpha L}}$, $V(\hat{\mathrm{P}}_i) = \frac{\sigma_{p_i}^2 + (n-1)0.25\sigma_{G_i}^2}{n}$, and $cov(\hat{\mathrm{P}}_i, \hat{\mathrm{P}}_k) = \frac{\sigma_{p_{ik}} + (n-1)0.25\sigma_{G_{ik}}}{n}$, where $\sigma_{p_i}^2$ is phenotypic variance of ith phenotypic RR growth curve coefficients, $\sigma_{G_i}^2$ is genetic variance of ith RR growth curve coefficients, and $\sigma_{G_{ik}}$ is genetic covariance of ith and k^{th} RR growth curve coefficients.

2. Phenotypic maximum growth index (I_p) based on the individual's own phenotypic RR growth curve coefficients

$I_p = \sum_{j=0}^{k-1} b_j P_j = \boldsymbol{bP},$

where P_j is the own phenotypic RR growth curve coefficients. Index weights ($\boldsymbol{b}$) can be obtained by iteration based on inversion the following matrix (2).

$$\begin{bmatrix} \boldsymbol{P}_{\alpha L} & \bar{\imath}\sqrt{\boldsymbol{b}'\boldsymbol{P}_{\alpha L}\boldsymbol{b}}\boldsymbol{G}_{\alpha L}\boldsymbol{S}' - \boldsymbol{P}_{\alpha L}\boldsymbol{b}\Delta\boldsymbol{G}'_s \\ \frac{\bar{\imath}}{\sqrt{\boldsymbol{b}'\boldsymbol{P}_{\alpha L}\,\boldsymbol{b}}}\boldsymbol{S}\boldsymbol{G}_{\alpha L} & \boldsymbol{0} \end{bmatrix} \begin{bmatrix} \boldsymbol{b} \\ \boldsymbol{\eta} \end{bmatrix} = \begin{bmatrix} \boldsymbol{G}_{\alpha L}\boldsymbol{F}' \\ \Delta\boldsymbol{G}_s \end{bmatrix} \quad (2)$$

, where $\boldsymbol{P}_{\alpha L}$ is a (k× k) phenotypic (co)variance matrix of P_j.

Note that $\boldsymbol{P}_{\alpha L}$ in (1) is (co)variance matrix of the average phenotypic RR growth curve coefficients of n progeny of a candidate bull and $\boldsymbol{P}_{\alpha L}$ in (2) is (co)variance matrix of the individual's own phenotypic RR growth curve coefficients.
